# Specialized active leprosy search strategies in an endemic area of the Brazilian Amazon identifies a hypermutated *Mycobacterium leprae* strain causing primary drug resistance

**DOI:** 10.3389/fmed.2023.1243571

**Published:** 2023-09-13

**Authors:** Raquel Carvalho Bouth, Angélica Rita Gobbo, Josafá Gonçalves Barreto, Pablo Diego do Carmo Pinto, Maraya Semblano Bittencourt, Marco Andrey Cipriani Frade, Apolônio Carvalho Nascimento, Sabrina Sampaio Bandeira, Patricia Fagundes da Costa, Guilherme Augusto Barros Conde, Charlotte Avanzi, Ândrea Ribeiro-dos-Santos, John Stewart Spencer, Moises Batista da Silva, Claudio Guedes Salgado

**Affiliations:** ^1^Laboratório de Dermato-Imunologia, Universidade Federal do Pará, Marituba, Pará, Brazil; ^2^Spatial Epidemiology Laboratory, Federal University of Pará, Castanhal, Brazil; ^3^Laboratório de Genética Humana e Médica, ICB, UFPA, Belém, Brazil; ^4^Santa Casa de Misericórdia do Pará– Serviço de Dermatologia– UFPA, Belém, Brazil; ^5^Divisão de Dermatologia, Departamento de Clínica Médica da Faculdade de Medicina de Ribeirão Preto, USP, Ribeirão Preto, São Paulo, Brazil; ^6^Unidade de Referência Especializada em Dermatologia Sanitária do Estado do Pará – URE Dr. Marcelo Candia, Marituba, Pará, Brazil; ^7^Laboratório de Suporte à Distância, Universidade Federal do Oeste do Pará, Santarém, Pará, Brazil; ^8^Department of Medical Parasitology and Infection Biology, Swiss Tropical and Public Health Institute, Basel, Switzerland; ^9^Department of Microbiology, Immunology and Pathology, Mycobacteria Research Laboratories, Colorado State University, Fort Collins, CO, United States; ^10^Coordenação de Atenção às Doenças Transmissíveis na Atenção Primária à Saúde, Departamento de Gestão do Cuidado Integral, Secretaria de Atenção Primária à Saúde, Ministério da Saúde, Brasília, Brazil

**Keywords:** leprosy, *Mycobacterium leprae*, household contacts, school children, drug resistance

## Abstract

**Introduction:**

Leprosy, an infectious disease caused by *Mycobacterium leprae*, remains a public health concern in endemic countries, particularly in Brazil. In this study, we conducted an active surveillance campaign in the hyperendemic city of Castanhal in the northeastern part of the state of Pará using clinical signs and symptoms combined with serological and molecular tools to diagnose new cases and to identify drug resistance of circulating *M. leprae* strains and their distribution in the community.

**Methods:**

During an active surveillance of one week, we enrolled 318 individuals using three different strategies to enroll subjects for this study: (i) an active survey of previously treated cases from 2006 to 2016 found in the Brazil National Notifiable Disease Information System database (*n* = 23) and their healthy household contacts (HHC) (*n* = 57); (ii) an active survey of school children (SC) from two primary public schools in low-income neighborhoods (*n* = 178), followed by visits to the houses of these newly diagnosed SC (*n* = 7) to examine their HHC (*n* = 34) where we diagnosed additional new cases (*n* = 6); (iii) and those people who spontaneously presented themselves to our team or the local health center with clinical signs and/or symptoms of leprosy (*n* = 6) with subsequent follow-up of their HHC when the case was confirmed (*n* = 20) where we diagnosed two additional cases (*n* = 2). Individuals received a dermato-neurological examination, 5 ml of peripheral blood was collected to assess the anti-PGL-I titer by ELISA and intradermal earlobe skin scrapings were taken from HHC and cases for amplification of the *M. leprae* RLEP region by qPCR.

**Results:**

Anti-PGL-I positivity was highest in the new leprosy case group (52%) followed by the treated group (40.9%), HHC (40%) and lowest in SC (24.6%). RLEP qPCR from SSS was performed on 124 individuals, 22 in treated cases, 24 in newly diagnosed leprosy cases, and 78 in HHC. We detected 29.0% (36/124) positivity overall in this sample set. The positivity in treated cases was 31.8% (7/22), while in newly diagnosed leprosy cases the number of positives were higher, 45.8% (11/23) and lower in HHC at 23.7% (18/76). Whole genome sequencing of *M. leprae* from biopsies of three infected individuals from one extended family revealed a hypermutated *M. leprae* strain in an unusual case of primary drug resistance while the other two strains were drug sensitive.

**Discussion:**

This study represents the extent of leprosy in an active surveillance campaign during a single week in the city of Castanhal, a city that we have previously surveyed several times during the past ten years. Our results indicate the continuing high transmission of leprosy that includes fairly high rates of new cases detected in children indicating recent spread by multiple foci of infection in the community. An unusual case of a hypermutated *M. leprae* strain in a case of primary drug resistance was discovered. It also revealed a high hidden prevalence of overt disease and subclinical infection that remains a challenge for correct clinical diagnosis by signs and symptoms that may be aided using adjunct laboratory tests, such as RLEP qPCR and anti-PGL-I serology.

## 1. Introduction

Leprosy, caused by the human pathogen *Mycobacterium leprae*, is a chronic, slowly evolving disease that causes damage to skin and nerves resulting in a wide array of skin lesions, nerve inflammation and pain leading to nerve impairment, loss of sensation, muscle weakening, atrophy and bone loss leading to disfigurement and disability with resulting social stigma. It remains a public health problem, especially in middle and low-income countries, such as India, Brazil, and Indonesia, where 79.6% of all global new cases were reported in 2019, when 202,185 new cases were detected globally. Brazil detected the second largest number of cases worldwide after India, with 27,863 new cases (1). The Brazil Amazon region, besides being highly endemic, has been depicted as having a very high hidden prevalence of leprosy (2). *M. leprae* primarily infects the peripheral nerves and later the skin (3, 4). Transmission from person to person is thought to be through the aerosol route, mainly in persons living in close contact for extended periods of time (5). Therefore, daily and continuous exposure with untreated patients make household contacts (HHC) a high-risk group in disease control strategies (6). Leprosy in children below 15 years old indicates recent infection to the bacillus during the early years of life and active circulation of bacilli in the community (7). This group was included as a target in the strategy for early detection and disrupting the transmission chain, aiming for the elimination of leprosy as a public health problem by the World Health Organization (8).

Early detection through contact tracing and active surveillance is essential to break the chain of transmission, to prevent severe neural involvement and physical disabilities due to disease progression. The diagnosis still relies on identifying well-characterized clinical signs and symptoms, with the detection of peripheral nerve damage, loss of sensation, and skin lesions. Laboratory tools, such as bacilloscopy in slit skin smears (SSS) (9), histopathology of skin lesions and molecular biology for detecting the *M. leprae*-specific repetitive element RLEP in SSS and skin biopsy (10, 11) as well as anti-PGL-I serology titer (2, 12, 13) support case elucidation, patient and HHC follow-up, and evaluation of subclinical infection in the community.

Together with the difficulties in the clinical diagnosis of leprosy and the absence of laboratory tools, drug resistance is an aggravating factor in controlling leprosy. The emergence of drug resistance has been reported since 1960 (14), and the presence of point mutations within genes in the drug resistance determining region (DRDR) is widely considered an important molecular signature for drug resistance in leprosy (15). Mutations in the *folP1* and *rpoB* genes confer resistance to the first line drugs used in the multidrug therapy (MDT) regimen, dapsone and rifampicin respectively, while mutations in *gyrA* and *gyrB* confer resistance to quinolones, second-line drugs of choice for leprosy treatment (16, 17).

Drug-resistant strains from 2009–2015 were recently described worldwide from MB leprosy cases from 19 sentinel countries for resistance to rifampicin, dapsone and ofloxacin showing around 2.3% in new cases and 4.5% in relapsed cases with 154 out of 1,932 (8%) *M. leprae* strains found overall with drug resistant mutations (18). In Brazil, a study with relapsed leprosy patients from the states of Rio de Janeiro, Espírito Santo, Amazonas, Pará and Ceará showed mutations associated with drug-resistance in *folP1* (5.3%), *rpoB* (7%), and *gyrA* (2.6%) (19). In the Brazilian Amazon region, the detection of drug resistance variants reached 43.2% among leprosy patients in a former leprosy colony, Prata Village (20), that is located less than 40 Km from Castanhal, the city of our study.

In this study, we conducted an active surveillance campaign in the hyperendemic city of Castanhal using clinical signs and symptoms combined with serological and molecular tools to diagnose new cases and to identify drug resistance of circulating *M. leprae* strains and their distribution in the community.

## 2. Methods

### 2.1. Study area

Castanhal is a municipality located 68 Km from Belém, the capital city of the state of Pará, Northern Brazil Amazon region, with an estimated population of 198,000 inhabitants in 2018 (21). The municipality has 76.4% of the urban population covered by the Family Health Strategy (SUS, the primary health service in charge of leprosy diagnosis), which is much higher than Belém that had only 22% coverage in 2016 (22). The average leprosy new case detection rate in the last ten years in Castanhal was 42.7/100,000 inhabitants, considered hyperendemic according to the WHO and Brazilian Ministry of Health (23).

### 2.2. Fieldwork

**Figure 1:**
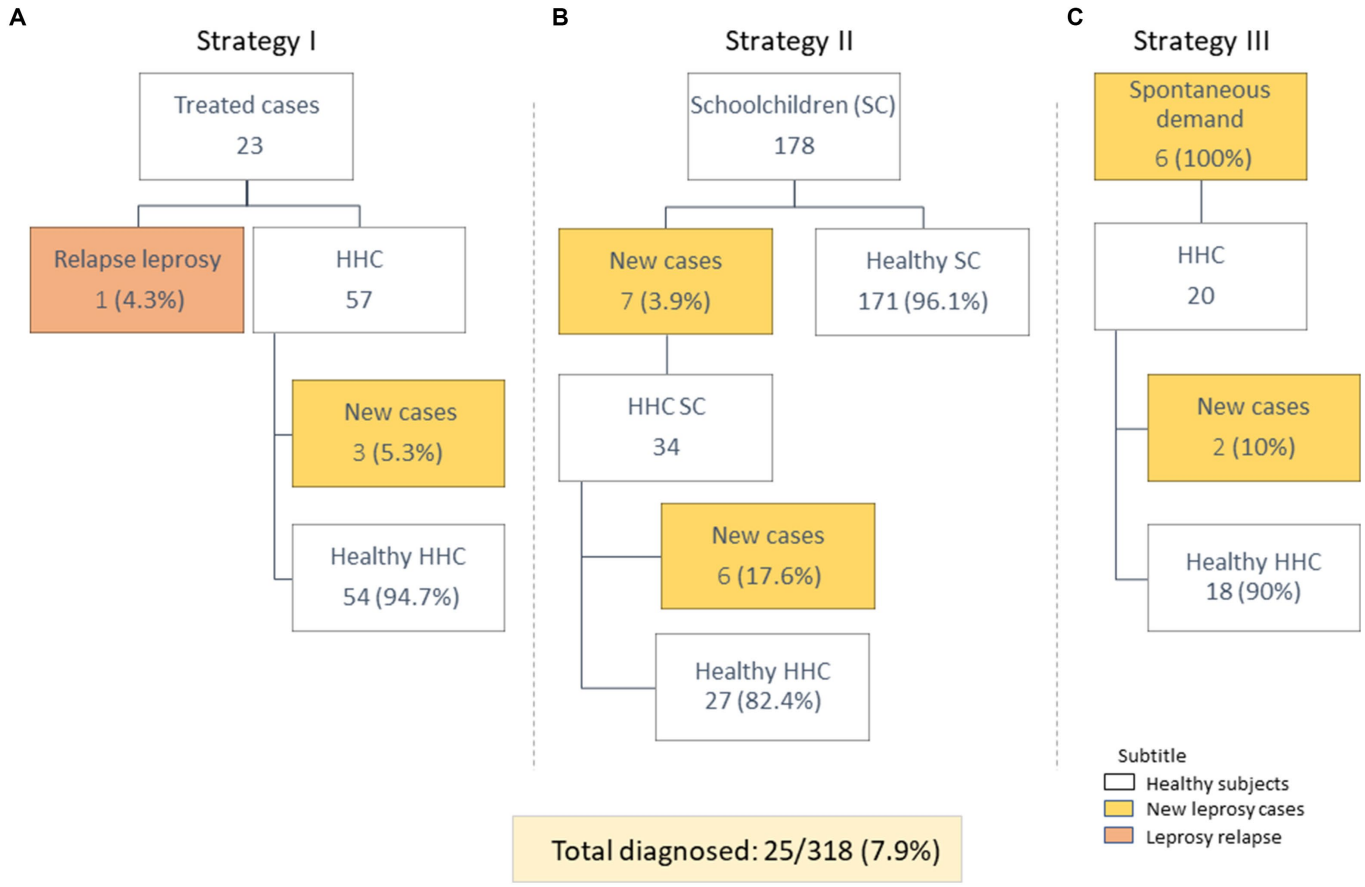
Distribution of study participants, according to each of the three active search strategies. **(A)** Strategy I: identified previously treated leprosy patients with follow-up with their HHC. **(B)** Strategy II: examined schoolchildren with follow-up of their HHC. **(C)** Strategy III: identified individuals who came to the clinic with suspected symptoms of leprosy with follow-up of their HHC.

Fieldwork was carried out using three distinct strategies: (i) active survey of reported and multidrug therapy (MDT) treated cases from 2006 to 2016 at the Brazil National Notifiable Disease Information System and their HHC (Figure 1A), (ii) active survey of school children (SC) from two primary public schools in peripheral and low-income neighborhoods, followed by a visit of the houses of the SC diagnosed with leprosy to examine their HHC (Figure 1B), and (iii) people who spontaneously presented themselves to our team, or the local health center, with signs and/or symptoms of leprosy as well as a visit to their HHC when the case was confirmed (Figure 1C).

The active survey was conducted according to the following scheme. All subjects were clinically evaluated by our team of health professionals (including a leprosy specialist, nurse and physiotherapist) and had peripheral blood and earlobe SSS collected according to established protocols. Biopsy of skin lesions was performed for pathological analyses by hematoxylin and eosin to detect cellular infiltrates and Fite-Faraco staining for quantifying acid-fast bacilli (AFB) (24) in a logarithmic index, resulting in the bacillary index (BI) registered from 0 to 6+ (25) depending on the number of AFB detected in the sample. The sample’s BI is related to the number of *M. leprae* genome copies in the sample collected which is a determining factor for predicting the success rate for *M. leprae* whole genome sequencing (WGS) (26).

### 2.3. Clinical evaluation

In the clinical evaluation, the leprosy physician examines the skin of each individual and when suspected characteristic skin lesions were detected, a sensitivity test was performed using the Semmes-Weinstein monofilament (27). Based on the Simplified Neurological Evaluation protocol proposed by the Brazil leprosy control program (28), peripheral nerves were examined by palpation as well as determining sensitivity, motor and autonomic functions for all nerves, including trigeminal, facial and auricular on face and neck; radial, radial cutaneous, median and ulnar nerves in the upper limbs; and fibular, superficial fibular and tibial nerves in the lower limbs. The assessment of neural impairment and grade of disability varied from 0 to 2, where grade 0 represents an absence of physical disability, grade 1 those individuals with decrease or loss of sensitivity on hands and/or feet, and grade 2 those with visible physical disabilities in eyes and/or limbs (8).

### 2.4. Laboratory analyses

Five milliliters of peripheral blood were collected from all individuals for the serological assay for the detection of anti-PGL-I antibodies by the ELISA technique, using the ND-O-HSA antigen, through a protocol described previously (29).

SSS were collected from both earlobes in one eppendorf tube containing 70% ethanol (13). After rehydration of the pelleted material in phosphate buffer saline (PBS), DNA extraction was performed using the Qiagen DNeasy Blood and Tissue Kit (Qiagen, Germantown, MD) following the manufacturer’s recommendations. Amplification of the specific *M. leprae* RLEP region was performed by quantitative PCR (qPCR) using forward LP1 (5′-GTGAGGGTAGTTGTT-3′) and reverse LP2 (5′-GGTGCGAATAGTT-3′) primers (30). The qPCR amplification mixture contained 5 μL of PCR grade water, 10 μL of SYBR green fluorescent DNA binding dye, 1 μL of primers and 10 ng of total DNA or 10 ng of positive control *M. leprae* DNA, or 4 μL of PCR grade water as a negative control, in a total volume of 25 μL per reaction. Each reaction was conducted in duplicate and the contents were processed and read by an Applied Biosystems® 7,500 Real-Time PCR System. The reaction occurred with the following specifications: Uracil-DNA glycosylase (UDG) at 50°C for 2 min, prior 95°C for 2 min for initial denaturation followed by 45 cycles, each cycle consisting of denaturation at 95°C for 15 s, annealing at 58°C for 15 s and extension at 72°C for 1 min. A melting curve was performed in each experiment. A standard amplification curve was prepared with purified *M. leprae* starting at 10^9^ bacilli genome copies/μL. The standard curve was composed of five points and was performed by serial dilution (1,100 to 1,5,000). The melting curve was used to analyze the specificity of the amplification. The results were obtained according to the first fluorescence signal detection cycle threshold (Ct). The sample was considered positive when duplicate samples showed a Ct less than 45 cycles. The standard curve was performed on each plate and included three negative control samples for each experiment.

Two skin biopsies were collected from each patient showing altered sensitivity skin lesions by a dermatologist using a 4 mm disposable punch (25). One fragment was stored in 10% formalin for histopathological examination and the other fragment was placed in 70% alcohol for WGS. Formalin fixed samples were dehydrated, clarified, and embedded in paraffin. Slides of 5 μm thickness were obtained from blocks sectioned with a microtome and subsequently deparaffinized. Sections were stained with hematoxylin–eosin to evaluate cellular infiltration and with Fite-Faraco for AFB detection (31).

For WGS of skin biopsy material, DNA was extracted using a pre-established protocol combining host tissue digestion and the QIAmp microbiome kit for host DNA depletion, strong bacterial cell lysis, and silica-based purification (26). Libraries with low *M. leprae* content underwent enrichment using whole-genome tiling arrays as described previously (32).

### 2.5. Statistical analysis

To compare the medians of the test results, the Mann–Whitney test was performed for two independent non-parametric samples. The statistical test and the plotting of results on graphs were performed using the GraphPad prism® program (version 6.1), the significance level of 0.5 (*p* ≤ 0.05) was used.

## 3. Results

During the fieldwork week, we evaluated a total of 318 individuals and diagnosed 25 cases (7.9%) using the three different strategies (Figure 1). In the previously treated case group, we evaluated 23 individuals and diagnosed one relapse (1/23; 4.3%). Among their HHC, three new cases were diagnosed (3/57; 5.3%) (Figure 1A). In the SC survey, 178 students were examined and 7 were diagnosed as new leprosy cases (7/178, 3.9%). The HHC of newly diagnosed SC were examined and six of 34 of these (17.6%) were diagnosed (Figure 1B). Six individuals with spontaneous demand (those who visited the clinic with symptoms of leprosy) were diagnosed (6/6; 100.0%) and two of the 20 HHC from these new cases (2/20; 10.0%) were diagnosed (Figure 1C).

The newly diagnosed leprosy cases (n = 25) ranged in age from 4 to 64 years old. Of these, nine (9/25; 36%) were children under 15 years old. The clinical forms were classified as: Primary neural (2/25; 8%), Indeterminate (3/25; 12%), Tuberculoid (1/25; 4%), Borderline (17/25; 60%), and Lepromatous leprosy (2/25; 8%). The disability grade of new cases was categorized as: Grade 0 (17/25; 68%), Grade 1 (6/25; 24%), and Grade 2 (2/25; 8%).

**Figure 2:**
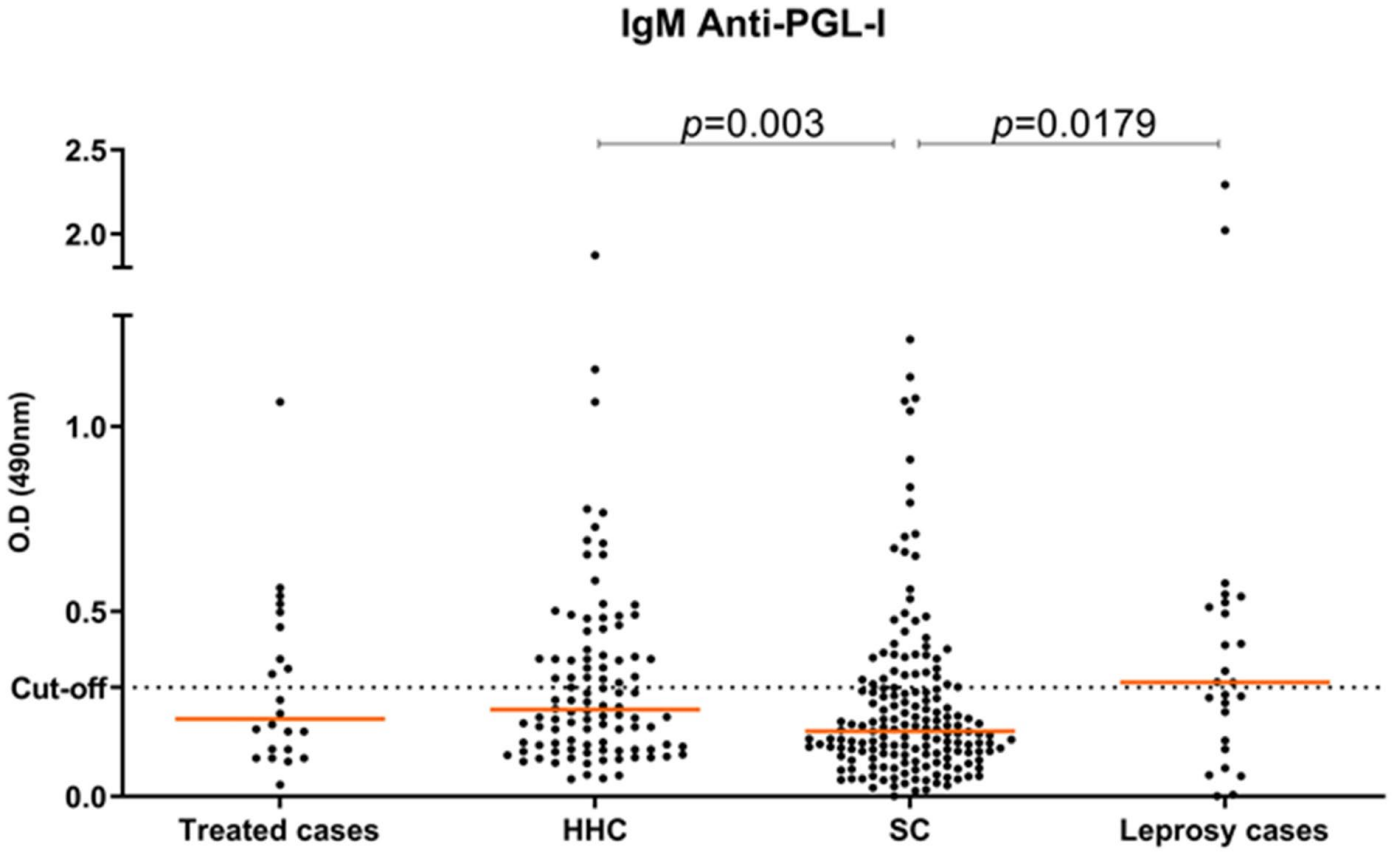
Titer of anti-PGL-I antibodies for all individuals according to study groups: treated cases (*n* = 22); HHC (*n* = 100); SC (*n* = 171); new leprosy cases (*n* = 25).

The anti-PGL-I IgM antibody titer was positive in 32.7% of all individuals (104/318). Among newly diagnosed leprosy cases, the positivity was 52% (13/25), the O.D. median was 0.31 while for treated cases the positivity was 40.9% (9/22) with an O.D. median of 0.21. HHC were positive in 40.0% (40/100) with an O.D. median of 0.24 and 24.6% (42/171) of SC were positive with an O.D. median of 0.18 (Table 1 and Supplementary Table S1). The statistical test showed a significant difference between SC and HHC (*p* = 0.003, 95% CI −0.09 to −0.019) and between SC and new leprosy cases (*p* = 0.018, 95% CI −0.017 to −0.198) (Figure 2).

**Table 1:** Positivity of anti-PGL-I IgM, molecular detection of RLEP and association of the two tests in the groups of the study.

Groups	Anti-PGL-I					RLEP (qPCR)				Double			
	Median	Positive		Negative		Positive		Negative		Positive		Negative	
	(O.D.)	(n)	(%)	(n)	(%)	(n)	(%)	(n)	(%)	(n)	(%)	(n)	(%)
New leprosy cases	0.31	13	52.0	12	48.0	11	45.8	13	54.2	4	16.7	5	20.8
Leprosy treated cases	0.21	9	40.9	13	59.1	7	31.8	15	68.2	1	4.5	7	31.8
HHC^a^	0.24	40	40.0	60	60.0	18	23.1	60	76.9	2	2.0	32	34.0
SC^b^	0.18	42	24.6	129	75.4	NA^c^	NA	NA	NA	NA	NA	NA	NA
Total		104	32.7	214	67.3	36	29.0	88	71.0	7	5.0	44	31.4

a HHC: Household contacts.

b SC: School children.

c NA: Not available.

RLEP qPCR from SSS was performed on 124 individuals, 22 in treated cases, 24 in newly diagnosed leprosy cases, and 78 in HHC. We detected 29.0% (36/124) positivity overall in this sample set. The positivity in treated cases was 31.8% (7/22), while in newly diagnosed leprosy cases the number of positives were higher, 45.8% (11/23) and lower in HHC at 23.7% (18/76). The percentage of double-positives overall (anti-PGL-I IgM+/RLEP qPCR+) was 5.6% (7/124). In the individual groups double positivity was 16.7% (4/24) for new leprosy cases, 4.5% (1/22) for treated cases and 2.6% (2/78) for HHC (Table 1).

A total of 22 skin biopsies were sampled from newly diagnosed leprosy patients. Three samples (3/22, 13.6%) were confirmed as leprosy by histopathology due to the presence of AFB. Three samples (3/22, 13.6%) were classified as superficial spongious dermatitis; three samples (3/22, 13.6%) were classified as granulomatous dermatitis and 13 (13/22, 59.2%) were classified as superficial perivascular dermatitis. RLEP qPCR was performed for 17 biopsies and was positive in seven of these (7/17, 41.2%), among which only three (42.8%) were positive for AFB and confirmed as leprosy by histopathology. Of the remaining samples, 2/7 (28.6%) were characterized as superficial perivascular dermatitis while the other 2/7 (28.6%) were characterized as granulomatous dermatitis.

Only five of the RLEP positive samples had enough bacillary DNA for WGS (n = 2) or to fully sequence the drug resistance determining region (DRDR) by PCR sequencing (n = 3). The two strains fully sequenced were covered 111 (patient 3702) and 57 times (patient 51447), respectively. 51447 was wild type (WT) for *rpoB*, *folp1*, *gyrA*, and *gyrB* while another was found to be a hypermutated *M. leprae* strain (3702), with multiple mutations in the DRDR genes *folp1* (P55L), *gyrA* (V731I) and *gyrB* (T503I). There were additional mutations found in a number of other genes, including *fadD9* (G796S), *ribD* (A63T), *pks4* (M14I) and *nth* (N142fs) (26). Raw genome sequences were deposited into the NCBI Sequence Read Archive (SRA) with biosample numbers SAMN07514430 (3702 or Br2016-15) and SAMN36810538 (Br51447). Both of these isolates were SNP type 4 N which predominates in this region (Supplementary Table S1). The remaining three samples were WT in *rpoB* and *folp1* and two were WT for *gyrA*. None of the three amplified the *gyrB* gene, so this gene could not be characterized.

### 3.1. Case findings of three diagnosed leprosy patients in a single extended family

#### 3.1.1. The primary multidrug-resistant leprosy case

**Figure 3:**
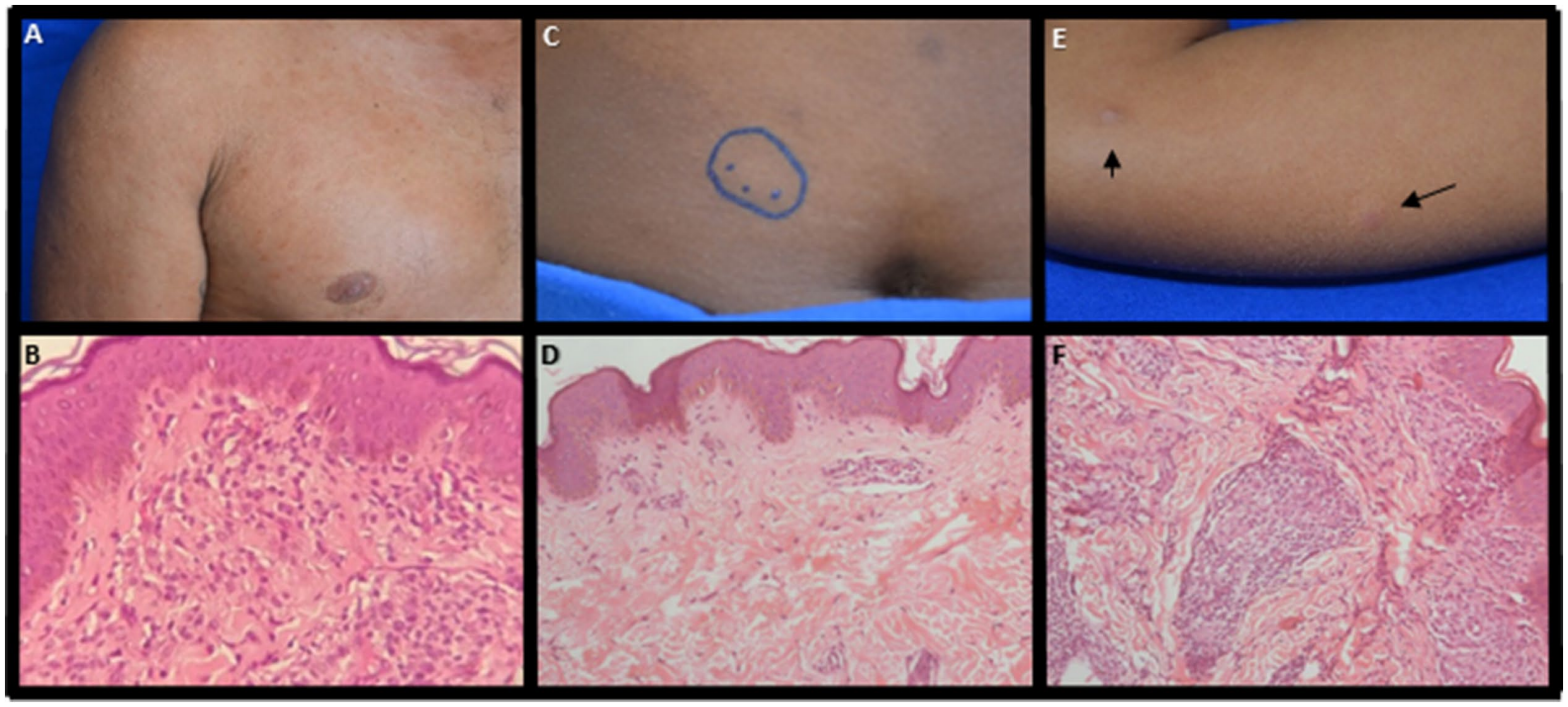
Clinical and pathological examination evaluation. Primary drug-resistant leprosy case **(A)** presence of infiltrative lesions and nodules disseminated through the integument; **(B)** dense granulomatous inflammatory infiltrate composed of lymphocytes, epithelioid histiocytes of foamy cytoplasm and plasmocytes, involving vessels, nerve filaments and superficial and deep plexus attachments; **(C)** spouse presented hypochromic plaque in the abdomen; **(D)** epidermis with a mild acanthosis and dermis with minimal perivascular lymphocytic infiltrate in the upper dermis and negative AFB. **(E)** son with hypochromic maculae with the presence of tubers in the right arm and elbow; **(F)** dense granulomatous inflammatory infiltrate of nodular architecture, composed of lymphocytes, plasmocytes and cytoplasmic epithelioid histiocytes with few positive AFB.

**Figure 4:**
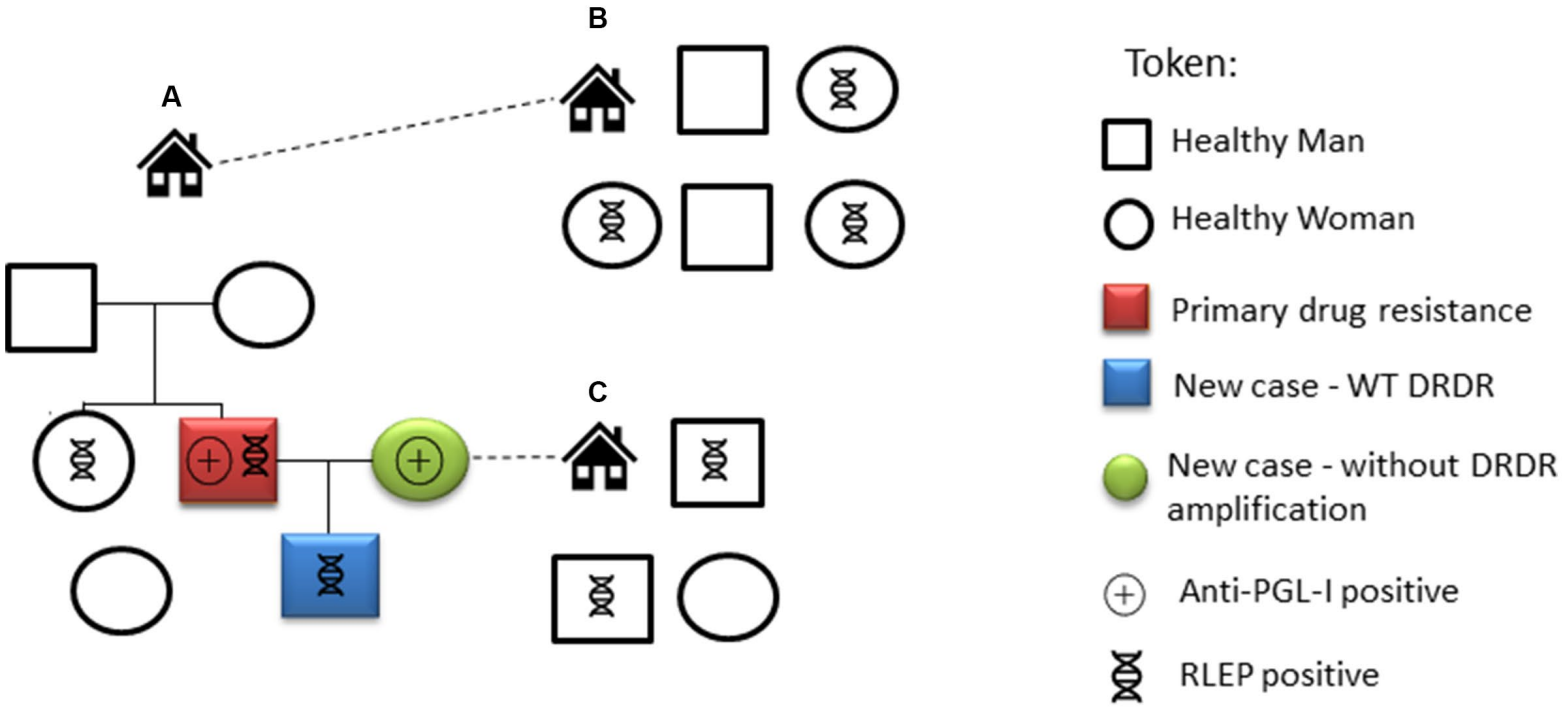
Evaluation and laboratory exams of the primary drug-resistant leprosy case and his contacts. House A residents: the individual primary drug-resistant case (red square), his spouse (green circle), their son (blue square) and other relatives. Residents of house B, located close to house A, are life-long contacts. Relatives of the spouse live in house C. The positive results for anti-PGL-I serology and detection of RLEP for each individual are shown.

A 31-year-old male with no prior history of leprosy presented infiltrative and nodular lesions disseminated throughout the skin, including face and ears, for at least one-year. After clinical evaluation, he was diagnosed with lepromatous leprosy (Figure 3A). The neurological evaluation showed three affected nerves with no disability (DG0). Adjunct laboratory tests demonstrated positive results for: SSS (BI 3.5), anti-PGL-I IgM antibody (O.D. = 2.02), positive RLEP qPCR in SSS (Ct = 32) and histopathological examination showing a dense superficial and deep granulomatous inflammatory infiltrate with a nodular architecture composed of lymphocytes, epithelioid histiocytes of foamy cytoplasm and plasmocytes involving vessels and nerve filaments (Figure 3B). The Fite-Faraco staining was also positive, with AFB either isolated or forming globi classified histopathologically as borderline lepromatous (BL) according to Ridley and Jopling classification. Molecular evaluation of the skin lesion was RLEP positive. Whole genome sequencing identified the strain as SNP subtype 4 N and as a hypermutated *M. leprae* strain with multiple mutations in the DRDR genes *folp1* (P55L), *gyrA* (V731I), *gyrB* (T503I), and in several other genes, including *fadD9* (G796S), *ribD* (A63T), *pks4* (M14I) and *nth* (N142fs). Regarding the treatment of this patient, after 11 doses of standard MDT, new nodular lesions in the lower limbs continued to appear at which time the WGS results confirmed dapsone resistance. The treatment regimen substituted daily minocycline 100 mg for dapsone and after 12 additional doses with this modified regimen the patient showed improvement in the clinical and laboratory parameters including an absence of active lesions, a decrease in the BI to 2.5 and a lower anti-PGL-I titer to 0.42. These laboratory parameters continued to decrease 6 months after medical discharge with a BI of 2.0 and a negative anti-PGL-I titer of 0.27. Fourteen of the HHC of this individual were evaluated, and 2 (14.3%), a spouse and son, were diagnosed with clinical signs and symptoms of leprosy. The IgM anti-PGL-I titers were positive only in 2 samples (14.3%) and negative in the remaining ten HHCs, varying from O.D. 0.11 to 0.28. The amplification of the RLEP of SSS samples by qPCR was positive in 6/12 HHC (50%) considered clinically healthy (Figure 4).

The spouse, 21-years-old, presented a hypochromic skin plaque larger than 10 cm diameter with imprecise edges (pseudopods) on the abdomen (Figure 3C) with loss of sensation, and DG0. She was classified as borderline leprosy (BT) with a positive anti-PGL-I titer (O.D. = 0.41) and negative for RLEP by qPCR in SSS. The histopathological examination of the skin biopsy showed an epidermis with mild acanthosis and minimal perivascular lymphocytic infiltrate in the upper dermis. AFB were absent by the Fite-Faraco stain, and the lesion was diagnosed as minimal superficial perivascular dermatitis (Figure 3D).

The son, 4-years-old, had hypochromic lesions with the presence of tubers in the left forearm, left forehead, and right forearm (Figure 3E) associated with thickening of the left ulnar nerve, DG0 and negative serology (O.D. = 0.28). He was also clinically classified as borderline leprosy. Histopathological examination demonstrated a dense granulomatous inflammatory infiltrate of nodular architecture composed of lymphocytes, plasmocytes and cytoplasmic epithelioid histiocytes (Figure 3F), with AFB (1+) on the sections examined by Fite-Faraco stain with the diagnostic definition of borderline tuberculoid (BT) leprosy by histopathology according to Ridley and Jopling classification. The qPCR performed on the SSS sample was positive (Ct = 41.4) for RLEP of *M. leprae*. The molecular analysis of the skin biopsy showed WT alleles in *rpoB*, *folp1*, *gyrA* and *gyrB*.

## 4. Discussion

Castanhal is a city in the northeastern part of Pará state, an endemic area that has been monitored by our leprosy surveillance team since 2010 (12). The municipality presents structural challenges in terms of public health, including the capacity to diagnose leprosy cases early and perform contact tracing and follow-up. In only 1 week of fieldwork, our group detected 25 new cases, which represents 71.4% of the number of cases detected in a year before the study (35 new cases) (29). The delay in diagnosis was supported by the presence of grade 2 physical disability (DG2) in 8% of cases and the number of new cases of leprosy in children under 15 (9/25, 36%) indicates ongoing recent infection from multiple foci of spread within the community corresponding to 4.5-fold more than was diagnosed by the local health team in 2015 (23). Leprosy diagnosis is primarily based on clinical signs and symptoms identified by well-trained leprologists. Laboratory tests with high sensitivity and specificity are not able to diagnose those with leprosy in all clinical forms and cannot even predict which at-risk HHC with positive anti-PGL-I titers will eventually progress to disease (33). However, laboratory tools may help identify biomarkers of subclinical infection, supported by the fact that individuals who do not show obvious clinical signs and symptoms of leprosy, considered healthy contacts, can be identified as having been infected if they have a positive anti-PGL-I titer and/or confirmed acid-fast bacilli or RLEP PCR positivity in SSS or skin lesion biopsy (13). We have previously shown that HHC with a positive anti-PGL-I titer have an 8.6-fold higher risk of progressing to disease than those with negative serology within 4 years (12). In this current study, almost 10% of the HHC had a confirmed leprosy diagnosis and 40.0% of clinically healthy HHC were seropositive. This means that 4 out of 10 HHC have this higher risk of developing leprosy.

Another important tool is the detection of *M. leprae* DNA, which may assist in the monitoring of asymptomatic HHC in an endemic area (34). In our study, we used RLEP, a repetitive region with up to 37 copies in the *M. leprae* genome (35). Therefore, its detection is efficient even when there are low levels of *M. leprae* DNA in different samples (10) and correlates with the bacilloscopy index and the clinical form (11). In our study, 23.1% of HHC had a positive RLEP qPCR result in SSS. In addition, we found that 2% of HHC were double-positive (anti-PGL-I+/RLEP qPCR+), results that we have previously established as likely representing latent leprosy disease (13). These individuals live in an endemic area, have leprosy cases in their household, are positive for *M. leprae* DNA in the ear lobe and show a non-protective immune response against the bacillus allowing its ability to grow and spread. Despite not showing clinical signs and symptoms of leprosy, individuals positive for both biomarkers of infection likely are subclinical with latent disease and need continuous monitoring by the local health team. Moreover, the presence of *M. leprae* confirmed by intradermal smear microscopy or skin biopsy is one of the cardinal signs for leprosy case definition by the WHO (36). In fact, RLEP qPCR is just a more sensitive method to detect *M. leprae* through the presence of DNA in either SSS or skin biopsy, and this alone should be considered sufficient to diagnose such individuals and to subsequently treat them early with MDT to effectively break the transmission chain and to avoid a delayed diagnosis with severe nerve damage and disability.

Our strategies of active surveillance for new cases among contacts of former patients that had already been treated and among school children allows many of these cases to be diagnosed in their earliest clinical manifestations, with light clinical signs and symptoms without significant nerve damage or disability, which are often poorly understood by the patient, their family and even for many untrained professionals. Thus, early diagnosis and treatment of cases prior to the development of nerve damage are extremely important to break the transmission chain and to avoid disfigurement and disabilities that can lead to stigma and social isolation.

The patient found with drug resistant *M. leprae* was apparently a case of primary drug resistance with no previous history of the patient being treated for leprosy. Luckily, our study showed that the son of this patient was not infected by this hypermutated strain, his strain was WT and drug sensitive. A limitation of this study was that although six of the 12 individuals in this extended family were RLEP+, none of these individuals had enough DNA to allow for sequencing and the spouse, who was diagnosed with leprosy, was qPCR negative for RLEP. Nevertheless, the finding of a patient with a strain resistant to dapsone, one of the main drugs used in the MDT regimen to treat most patients, in addition to mutations in *gyrA* and *gyrB* indicating possible resistance to fluoroquinolones, important second-line drugs used for the treatment of leprosy, should draw attention to the increased danger and prevalence of multidrug resistant strains and provide an incentive for increased funding for testing more clinical strains for drug resistance, especially in endemic areas. There is also a need to seek new alternative drug regimens that can be substituted in cases of resistance to the three main drugs used in MDT as was eventually used to treat the patient with the hypermutated strain and to identify new and more effective antimycobacterial drugs to facilitate a real break in the transmission chain of these strains in the community (37).

## 5. Conclusion

Our surveillance activities in just 1 week in an area hyperendemic for leprosy in the Amazon region of Brazil (Castanhal, Pará State) showed high transmission rates of leprosy. It also revealed a high hidden prevalence of overt disease and subclinical infection that remains a challenge for correct clinical diagnosis by signs and symptoms that may be aided using adjunct laboratory tests, such as RLEP qPCR and anti-PGL-I serology. The spread of leprosy can be worsened by the presence of drug resistant *M. leprae* strains that are potentially circulating in this population, which should be monitored more closely.

## Data availability statement

The datasets presented in this study can be found in online repositories. The names of the repository/repositories and accession number(s) can be found at (NCBI Sequence Read Archive, SRR6241736).

## Ethics statement

The studies involving human participants were reviewed and approved by Institute of Health Sciences Research Ethics Committee from Pará Federal University (CAAE 26765414.0.0000.0018 CEP-ICS/UFPA). Written informed consent to participate in this study was provided by the participant or participants’ legal guardian/next of kin. The studies were conducted in accordance with the local legislation and institutional requirements. Written informed consent was obtained from all individuals and/or minors’ legal guardian/next of kin for the publication of any potentially identifiable images or data included in this article, including data in Supplementary Table S1.

## Author contributions

RB, JB, JS, MS, and CS: contributed to the research design and writing the draft manuscript. RB, AG, MB, PCa, MB, MF, AN, SB, PCo, GC, ÂR-d-S, and CS: collected data and organized the database. RB, GC, CA, JS, MS, and CS: performed statistical and data analysis. All authors contributed to the article and approved the submitted version.

## Funding

This work was supported by CNPq (486183/2013–0 CNPq grant for MS; 448741/2014–8 grant for JB; 428964/2016–8 grant and 313633/2018–5 fellowship for CS; and 306815/2018–4 grant for ÂR-d-S), CAPES PROAMAZONIA 3288/2013, CAPES Biocomputacional – RPGPH (3381/2013), Brazil Ministry of Health 035527/2017, PROPESP/UFPA, VALE S.A. 27756/2019, Fulbright Scholar to Brazil 2019–2020 (JS) and the Heiser Program of the New York Community Trust for Research in Leprosy (JB, MS, CS, and JS) grants P15-000827, P16-000796, and P18-000250.

## Conflict of interest

The authors declare that the research was conducted in the absence of any commercial or financial relationships that could be construed as a potential conflict of interest.

## Publisher’s note

All claims expressed in this article are solely those of the authors and do not necessarily represent those of their affiliated organizations, or those of the publisher, the editors and the reviewers. Any product that may be evaluated in this article, or claim that may be made by its manufacturer, is not guaranteed or endorsed by the publisher.
