# Supplementary material for: Specialized active leprosy search strategies in an endemic area of the Brazilian Amazon identifies a hypermutated Mycobacterium leprae strain causing primary drug resistance
Source: Front Med (Lausanne). 2023 Sep 13;10:1243571. doi: 10.3389/fmed.2023.1243571 (PMC10534026; doi:10.3389/fmed.2023.1243571)
Supplement: Supplementary file 1 [file Table_1.doc]

Supplementary Table 1: Results of the clinical evaluation, anti-PGL-I serology (positive in bold), SSS RLEP qPCR, histopathological examination of the lesion, skin RLEP qPCR, *M. leprae* SNP and MDT sensitivity of all participants

|  | ID | Age | Anti-PGL-I  (O.D.) | SSS RLEP qPCR  (Ct) | New case | Clinical form | Disability grade | Histopatology diagnosis | *Fite-Faraco (AFB)* | Skin RLEP PCR | SNP | MDT sensitivity |
| --- | --- | --- | --- | --- | --- | --- | --- | --- | --- | --- | --- | --- |
| 1 | 3703 | 4 | 0.28 | 41.45 | Yes | BT | 0 | Leprosy BT | Pos | Pos | NA | susceptible |
| 2 | 3678 | 8 | 0.01 | Undetermined | Yes | BT | 0 | Superficial spongiotic dermatitis | Neg | NA | NA | NA |
| 3 | 3718 | 8 | 0.05 | 38.26 | Yes | BT | 0 | Granulomatous dermatitis | Neg | NA | NA | NA |
| 4 | 3685 | 11 | **0.50** | NA | Yes | BT | 1 | Superficial spongiotic dermatitis | Neg | NA | NA | NA |
| 5 | 3717 | 11 | **0.54** | 41.57 | Yes | I | 0 | Superficial perivascular dermatitis | Neg | NA | NA | NA |
| 6 | 3720 | 12 | 0.23 | Undetermined | Yes | BT | 0 | Superficial perivascular dermatitis | Neg | NA | NA | NA |
| 7 | 3670 | 13 | **0.31** | Undetermined | Yes | BT | 1 | Superficial and interstitial spongiotic dermatitis | NA | Neg | NA | NA |
| 8 | 3679 | 14 | **0.41** | Undetermined | Yes | BT | 0 | Superficial perivascular dermatitis | Neg | NA | NA | NA |
| 9 | 3684 | 14 | **0.51** | Undetermined | Yes | BT | 0 | Superficial perivascular dermatitis | Neg | NA | NA | NA |
| 10 | 3665 | 15 | 0.00 | Undetermined | Yes | BT | 0 | NA | NA | Neg | NA | NA |
| 11 | 3677 | 21 | **0.41** | Undetermined | Yes | BT | 0 | Superficial perivascular dermatitis | Neg | Neg | NA | NA |
| 12 | 3710 | 21 | 0.27 | 39.16 | Yes | PNL | 0 | Superficial perivascular dermatitis | Neg | Pos | NA | NA |
| 13 | 3682 | 24 | **0.34** | Undetermined | Yes | BT | 1 | Superficial perivascular dermatitis | Neg | Neg | NA | NA |
| 14 | 3702 | 31 | **2.02** | 32.00 | Yes | LL | 1 | LL | Pos | Pos | 4N | resistant |
| 15 | 3713 | 32 | 0.25 | Undetermined | Yes | PNL | 2 | Superficial perivascular dermatitis | Neg | Neg | NA | NA |
| 16 | 3680 | 34 | **0.55** | 36.15 | Yes | BT | 0 | Superficial perivascular dermatitis | Neg | Neg | NA | NA |
| 17 | 3707 | 34 | 0.27 | 37.19 | Yes | I | 0 | Superficial perivascular dermatitis | Neg | Neg | NA | NA |
| 18 | 50951 | 34 | 0.06 | 39.70 | Yes | BT | 0 | Granulomatous dermatitis | Neg | Pos | NA | susceptible |
| 19 | 51447 | 38 | **2.29** | 28.60 | Yes | BL | 2 | Leprosy | Pos | Pos | 4N | susceptible |
| 20 | 50999 | 41 | 0.13 | 42.90 | Yes | TT | 0 | Chronic granulomatous dermatitis | Neg | Pos | NA | susceptible |
| 21 | 3721 | 47 | 0.08 | 41.02 | Yes | BT | 1 | NA | NA | Neg | NA | NA |
| 22 | 3708 | 48 | **0.52** | Undetermined | Yes | BT | 0 | Superficial perivascular dermatitis | Neg | Neg | NA | NA |
| 23 | 3716 | 49 | 0.15 | Undetermined | Yes | I | 0 | Superficial perivascular dermatitis | Neg | Pos | NA | NA |
| 24 | 3719 | 52 | **0.31** | Undetermined | Yes | BT | 1 | Superficial perivascular dermatitis | Neg | Neg | NA | NA |
| 25 | 3705 | 64 | **0.58** | Undetermined | Yes | BT | 0 | NA | NA | NA | NA | NA |
| 26 | 231 | 88 | 0.03 | Undetermined | No | LL | 0 | NA | NA | NA | NA | NA |
| 27 | 214 | 40 | 0.09 | 36.96 | No | BB | 0 | NA | NA | NA | NA | NA |
| 28 | 259 | 66 | 0.10 | Undetermined | No | T | 0 | NA | NA | NA | NA | NA |
| 29 | 3704 | 53 | 0.10 | Undetermined | No | NA | 0 | NA | NA | NA | NA | NA |
| 30 | 203 | 40 | 0.10 | 40.79 | No | BT | 0 | NA | NA | NA | NA | NA |
| 31 | 232 | 59 | 0.13 | Undetermined | No | I | 0 | NA | NA | NA | NA | NA |
| 32 | 3655 | 54 | 0.13 | Undetermined | No | NA | 0 | NA | NA | NA | NA | NA |
| 33 | 279 | 53 | 0.18 | 40.21 | No | BT | 0 | NA | NA | NA | NA | NA |
| 34 | 3667 | nd | 0.18 | Undetermined | No | NA | 0 | NA | NA | NA | NA | NA |
| 35 | 281 | 27 | 0.18 | 40.42 | No | BT | 2 | NA | NA | NA | NA | NA |
| 36 | 213 | 39 | 0.20 | 30.85 | No | BT | 1 | NA | NA | NA | NA | NA |
| 37 | 3664 | 53 | 0.22 | Undetermined | No | NA | 0 | NA | NA | NA | NA | NA |
| 38 | 243 | 40 | 0.26 | 43.33 | No | BT | 0 | NA | NA | NA | NA | NA |
| 39 | 257 | 13 | **0.33** | 44.05 | No | BT | 0 | NA | NA | NA | NA | NA |
| 40 | 3656 | 15 | **0.35** | Undetermined | No | NA | 0 | NA | NA | NA | NA | NA |
| 41 | 244 | 41 | **0.37** | Undetermined | No | I | 0 | NA | NA | NA | NA | NA |
| 42 | 3659 | 25 | **0.46** | Undetermined | No | NA | 0 | NA | NA | NA | NA | NA |
| 43 | 247 | 19 | **0.50** | Undetermined | No | I | 0 | NA | NA | NA | NA | NA |
| 44 | 3653 | nd | **0.52** | Undetermined | No | NA | 0 | NA | NA | NA | NA | NA |
| 45 | 3651 | 20 | **0.54** | Undetermined | No | NA | 0 | NA | NA | NA | NA | NA |
| 46 | 3658 | nd | **0.56** | Undetermined | No | NA | 0 | NA | NA | NA | NA | NA |
| 47 | 290 | 37 | **1.07** | Undetermined | No | NA | 0 | NA | NA | NA | NA | NA |
| 48 | 217D | 47 | 0.15 | Undetermined | No | NA | NA | NA | NA | NA | NA | NA |
| 49 | 227F | 19 | 0.13 | Undetermined | No | NA | NA | NA | NA | NA | NA | NA |
| 50 | 227G | 12 | 0.20 | Undetermined | No | NA | NA | NA | NA | NA | NA | NA |
| 51 | 227H | 13 | 0.22 | Undetermined | No | NA | NA | NA | NA | NA | NA | NA |
| 52 | 227I | 3 | 0.28 | NA | No | NA | NA | NA | NA | NA | NA | NA |
| 53 | 231D | 56 | 0.11 | 31.87 | No | NA | NA | NA | NA | NA | NA | NA |
| 54 | 232H | 7 | 0.10 | NA | No | NA | NA | NA | NA | NA | NA | NA |
| 55 | 232I | 35 | 0.11 | Undetermined | No | NA | NA | NA | NA | NA | NA | NA |
| 56 | 232G | 36 | 0.25 | 40.01 | No | NA | NA | NA | NA | NA | NA | NA |
| 57 | 232F | 31 | **0.46** | Undetermined | No | NA | NA | NA | NA | NA | NA | NA |
| 58 | 243D | 15 | 0.18 | Undetermined | No | NA | NA | NA | NA | NA | NA | NA |
| 59 | 247F | 25 | 0.12 | Undetermined | No | NA | NA | NA | NA | NA | NA | NA |
| 60 | 247I | 72 | 0.15 | Undetermined | No | NA | NA | NA | NA | NA | NA | NA |
| 61 | 247G | 17 | 0.26 | Undetermined | No | NA | NA | NA | NA | NA | NA | NA |
| 62 | 247J | 41 | 0.28 | Undetermined | No | NA | NA | NA | NA | NA | NA | NA |
| 63 | 259E | 35 | **0.37** | Undetermined | No | NA | NA | NA | NA | NA | NA | NA |
| 64 | 920A | 46 | 0.18 | Undetermined | No | NA | NA | NA | NA | NA | NA | NA |
| 65 | 871A | 39 | 0.14 | Undetermined | No | NA | NA | NA | NA | NA | NA | NA |
| 66 | 871J | 23 | 0.19 | Undetermined | No | NA | NA | NA | NA | NA | NA | NA |
| 67 | 871E | 54 | 0.21 | Undetermined | No | NA | NA | NA | NA | NA | NA | NA |
| 68 | 871K | 38 | 0.23 | Undetermined | No | NA | NA | NA | NA | NA | NA | NA |
| 69 | 871D | 17 | 0.24 | Undetermined | No | NA | NA | NA | NA | NA | NA | NA |
| 70 | 871G | 10 | 0.29 | Undetermined | No | NA | NA | NA | NA | NA | NA | NA |
| 71 | 871F | 20 | **0.32** | Undetermined | No | NA | NA | NA | NA | NA | NA | NA |
| 72 | 871C | 9 | **0.50** | Undetermined | No | NA | NA | NA | NA | NA | NA | NA |
| 73 | 871H | 8 | **0.69** | Undetermined | No | NA | NA | NA | NA | NA | NA | NA |
| 74 | 871I | 50 | **0.77** | Undetermined | No | NA | NA | NA | NA | NA | NA | NA |
| 75 | 871B | 48 | **1.07** | Undetermined | No | NA | NA | NA | NA | NA | NA | NA |
| 76 | 3653A | 40 | 0.06 | Undetermined | No | NA | NA | NA | NA | NA | NA | NA |
| 77 | 3653C | 41 | 0.10 | Undetermined | No | NA | NA | NA | NA | NA | NA | NA |
| 78 | 3653B | 27 | **0.52** | NA | No | NA | NA | NA | NA | NA | NA | NA |
| 79 | 890D | 9 | **0.65** | Undetermined | No | NA | NA | NA | NA | NA | NA | NA |
| 80 | 831A | 65 | 0.13 | Undetermined | No | NA | NA | NA | NA | NA | NA | NA |
| 81 | 831C | 20 | **0.52** | Undetermined | No | NA | NA | NA | NA | NA | NA | NA |
| 82 | 831B | 48 | **0.65** | Undetermined | No | NA | NA | NA | NA | NA | NA | NA |
| 83 | 853B | nd | 0.09 | 39.00 | No | NA | NA | NA | NA | NA | NA | NA |
| 84 | 853D | 61 | **0.32** | 44.00 | No | NA | NA | NA | NA | NA | NA | NA |
| 85 | 853C | nd | **0.37** | Undetermined | No | NA | NA | NA | NA | NA | NA | NA |
| 86 | 853A | nd | **0.69** | Undetermined | No | NA | NA | NA | NA | NA | NA | NA |
| 87 | 3703D | 48 | 0.14 | 38.90 | No | NA | NA | NA | NA | NA | NA | NA |
| 88 | 3703C | 34 | 0.24 | 40.72 | No | NA | NA | NA | NA | NA | NA | NA |
| 89 | 3703B | 23 | 0.26 | 36.08 | No | NA | NA | NA | NA | NA | NA | NA |
| 90 | 3678A | 33 | **0.78** | Undetermined | No | NA | NA | NA | NA | NA | NA | NA |
| 91 | 3679C | 15 | 0.22 | Undetermined | No | NA | NA | NA | NA | NA | NA | NA |
| 92 | 3679B | 17 | **0.45** | Undetermined | No | NA | NA | NA | NA | NA | NA | NA |
| 93 | 3681A | 26 | **0.40** | Undetermined | No | NA | NA | NA | NA | NA | NA | NA |
| 94 | 3684A | 37 | 0.21 | Undetermined | No | NA | NA | NA | NA | NA | NA | NA |
| 95 | 3685A | 38 | 0.20 | Undetermined | No | NA | NA | NA | NA | NA | NA | NA |
| 96 | 3702F | 31 | 0.12 | 37.43 | No | NA | NA | NA | NA | NA | NA | NA |
| 97 | 3702D | 60 | 0.12 | 37.37 | No | NA | NA | NA | NA | NA | NA | NA |
| 98 | 3702G | 41 | 0.15 | 38.72 | No | NA | NA | NA | NA | NA | NA | NA |
| 99 | 3702J | 70 | 0.18 | Undetermined | No | NA | NA | NA | NA | NA | NA | NA |
| 100 | 3702B | 76 | 0.19 | 36.05 | No | NA | NA | NA | NA | NA | NA | NA |
| 101 | 3702H | 14 | 0.19 | 37.33 | No | NA | NA | NA | NA | NA | NA | NA |
| 102 | 3702E | 47 | 0.20 | Undetermined | No | NA | NA | NA | NA | NA | NA | NA |
| 103 | 3702I | 4 | 0.23 | 40.16 | No | NA | NA | NA | NA | NA | NA | NA |
| 104 | 3702A | 21 | 0.28 | 42.15 | No | NA | NA | NA | NA | NA | NA | NA |
| 105 | 3704A | 62 | 0.12 | NA | No | NA | NA | NA | NA | NA | NA | NA |
| 106 | 3704B | 7 | **0.32** | NA | No | NA | NA | NA | NA | NA | NA | NA |
| 107 | 3704C | 21 | **0.37** | NA | No | NA | NA | NA | NA | NA | NA | NA |
| 108 | 3706C | 14 | **0.45** | Undetermined | No | NA | NA | NA | NA | NA | NA | NA |
| 109 | 3709G | 14 | 0.14 | Undetermined | No | NA | NA | NA | NA | NA | NA | NA |
| 110 | 3709A | 51 | 0.14 | 39.72 | No | NA | NA | NA | NA | NA | NA | NA |
| 111 | 3709C | 12 | **0.30** | Undetermined | No | NA | NA | NA | NA | NA | NA | NA |
| 112 | 3709F | 19 | **0.38** | Undetermined | No | NA | NA | NA | NA | NA | NA | NA |
| 113 | 3709D | 18 | **0.73** | Undetermined | No | NA | NA | NA | NA | NA | NA | NA |
| 114 | 3709B | 50 | **1.87** | Undetermined | No | NA | NA | NA | NA | NA | NA | NA |
| 115 | 3711A | 45 | **0.48** | Undetermined | No | NA | NA | NA | NA | NA | NA | NA |
| 116 | 3712B | 56 | 0.06 | Undetermined | No | NA | NA | NA | NA | NA | NA | NA |
| 117 | 3712C | 36 | **0.38** | Undetermined | No | NA | NA | NA | NA | NA | NA | NA |
| 118 | 3712A | 42 | **1.16** | Undetermined | No | NA | NA | NA | NA | NA | NA | NA |
| 119 | 3713B | 38 | **0.32** | NA | No | NA | NA | NA | NA | NA | NA | NA |
| 120 | 3713A | 37 | **0.32** | 43.00 | No | NA | NA | NA | NA | NA | NA | NA |
| 121 | 3714C | 70 | 0.11 | Undetermined | No | NA | NA | NA | NA | NA | NA | NA |
| 122 | 3714B | 62 | 0.11 | 43.00 | No | NA | NA | NA | NA | NA | NA | NA |
| 123 | 3714A | 32 | **0.49** | Undetermined | No | NA | NA | NA | NA | NA | NA | NA |
| 124 | 3715A | 34 | 0.21 | Undetermined | No | NA | NA | NA | NA | NA | NA | NA |
| 125 | 3715C | 9 | 0.22 | NA | No | NA | NA | NA | NA | NA | NA | NA |
| 126 | 3715D | 27 | **0.35** | Undetermined | No | NA | NA | NA | NA | NA | NA | NA |
| 127 | 3715B | 54 | **0.37** | Undetermined | No | NA | NA | NA | NA | NA | NA | NA |
| 128 | 3716B | 54 | 0.05 | Undetermined | No | NA | NA | NA | NA | NA | NA | NA |
| 129 | 3716A | 26 | 0.09 | Undetermined | No | NA | NA | NA | NA | NA | NA | NA |
| 130 | 3717B | 16 | **0.49** | Undetermined | No | NA | NA | NA | NA | NA | NA | NA |
| 131 | 3717A | 41 | **0.58** | Undetermined | No | NA | NA | NA | NA | NA | NA | NA |
| 132 | 3718A | 31 | 0.05 | NA | No | NA | NA | NA | NA | NA | NA | NA |
| 133 | 3718B | 14 | 0.10 | NA | No | NA | NA | NA | NA | NA | NA | NA |
| 134 | 3718C | 33 | **0.31** | NA | No | NA | NA | NA | NA | NA | NA | NA |
| 135 | 3719A | 76 | 0.09 | NA | No | NA | NA | NA | NA | NA | NA | NA |
| 136 | 3719G | 11 | 0.14 | NA | No | NA | NA | NA | NA | NA | NA | NA |
| 137 | 3719F | 32 | 0.21 | NA | No | NA | NA | NA | NA | NA | NA | NA |
| 138 | 3719E | 47 | **0.35** | NA | No | NA | NA | NA | NA | NA | NA | NA |
| 139 | 3719I | 35 | **0.37** | NA | No | NA | NA | NA | NA | NA | NA | NA |
| 140 | 3719B | 28 | **0.37** | NA | No | NA | NA | NA | NA | NA | NA | NA |
| 141 | 3719H | 27 | **0.48** | NA | No | NA | NA | NA | NA | NA | NA | NA |
| 142 | 3719C | 19 | **0.49** | NA | No | NA | NA | NA | NA | NA | NA | NA |
| 143 | 3721C | 11 | 0.10 | NA | No | NA | NA | NA | NA | NA | NA | NA |
| 144 | 3721A | 78 | 0.11 | NA | No | NA | NA | NA | NA | NA | NA | NA |
| 145 | 3721B | 8 | 0.13 | NA | No | NA | NA | NA | NA | NA | NA | NA |
| 146 | 50951A | 21 | 0.26 | 44.30 | No | NA | NA | NA | NA | NA | NA | NA |
| 147 | 50999A | nd | 0.13 | Undetermined | No | NA | NA | NA | NA | NA | NA | NA |
| 148 | E8040 | 14 | 0.00 | NA | No | NA | NA | NA | NA | NA | NA | NA |
| 149 | E8230 | 7 | 0.02 | NA | No | NA | NA | NA | NA | NA | NA | NA |
| 150 | E8142 | 10 | 0.02 | NA | No | NA | NA | NA | NA | NA | NA | NA |
| 151 | E8221 | 10 | 0.02 | NA | No | NA | NA | NA | NA | NA | NA | NA |
| 152 | E8004 | 11 | 0.03 | NA | No | NA | NA | NA | NA | NA | NA | NA |
| 153 | E8209 | 11 | 0.03 | NA | No | NA | NA | NA | NA | NA | NA | NA |
| 154 | E8052 | 11 | 0.04 | NA | No | NA | NA | NA | NA | NA | NA | NA |
| 155 | E8057 | 13 | 0.04 | NA | No | NA | NA | NA | NA | NA | NA | NA |
| 156 | E8098 | 10 | 0.04 | NA | No | NA | NA | NA | NA | NA | NA | NA |
| 157 | E8214 | 10 | 0.04 | NA | No | NA | NA | NA | NA | NA | NA | NA |
| 158 | E8200 | 12 | 0.05 | NA | No | NA | NA | NA | NA | NA | NA | NA |
| 159 | E8229 | 11 | 0.05 | NA | No | NA | NA | NA | NA | NA | NA | NA |
| 160 | E8071 | 14 | 0.05 | NA | No | NA | NA | NA | NA | NA | NA | NA |
| 161 | E8201 | 11 | 0.05 | NA | No | NA | NA | NA | NA | NA | NA | NA |
| 162 | E8210 | 9 | 0.05 | NA | No | NA | NA | NA | NA | NA | NA | NA |
| 163 | E8082 | 11 | 0.05 | NA | No | NA | NA | NA | NA | NA | NA | NA |
| 164 | E8118 | 15 | 0.05 | NA | No | NA | NA | NA | NA | NA | NA | NA |
| 165 | E8006 | 12 | 0.05 | NA | No | NA | NA | NA | NA | NA | NA | NA |
| 166 | E8143 | 8 | 0.06 | NA | No | NA | NA | NA | NA | NA | NA | NA |
| 167 | E8130 | 13 | 0.06 | NA | No | NA | NA | NA | NA | NA | NA | NA |
| 168 | E8113 | 13 | 0.06 | NA | No | NA | NA | NA | NA | NA | NA | NA |
| 169 | E8091 | 14 | 0.06 | NA | No | NA | NA | NA | NA | NA | NA | NA |
| 170 | E8226 | 15 | 0.07 | NA | No | NA | NA | NA | NA | NA | NA | NA |
| 171 | E8133 | 11 | 0.07 | NA | No | NA | NA | NA | NA | NA | NA | NA |
| 172 | E8090 | 13 | 0.07 | NA | No | NA | NA | NA | NA | NA | NA | NA |
| 173 | E8029 | 12 | 0.08 | NA | No | NA | NA | NA | NA | NA | NA | NA |
| 174 | E8086 | 10 | 0.08 | NA | No | NA | NA | NA | NA | NA | NA | NA |
| 175 | E8009 | 13 | 0.08 | NA | No | NA | NA | NA | NA | NA | NA | NA |
| 176 | E8060 | 12 | 0.08 | NA | No | NA | NA | NA | NA | NA | NA | NA |
| 177 | E8134 | 11 | 0.08 | NA | No | NA | NA | NA | NA | NA | NA | NA |
| 178 | E8223 | 9 | 0.08 | NA | No | NA | NA | NA | NA | NA | NA | NA |
| 179 | E8216 | 11 | 0.08 | NA | No | NA | NA | NA | NA | NA | NA | NA |
| 180 | E8067 | 12 | 0.09 | NA | No | NA | NA | NA | NA | NA | NA | NA |
| 181 | E8070 | 14 | 0.09 | NA | No | NA | NA | NA | NA | NA | NA | NA |
| 182 | E8100 | 10 | 0.09 | NA | No | NA | NA | NA | NA | NA | NA | NA |
| 183 | E8126 | 14 | 0.10 | NA | No | NA | NA | NA | NA | NA | NA | NA |
| 184 | E8218 | 14 | 0.10 | NA | No | NA | NA | NA | NA | NA | NA | NA |
| 185 | E8117 | 16 | 0.10 | NA | No | NA | NA | NA | NA | NA | NA | NA |
| 186 | E8093 | 11 | 0.10 | NA | No | NA | NA | NA | NA | NA | NA | NA |
| 187 | E8119 | 11 | 0.10 | NA | No | NA | NA | NA | NA | NA | NA | NA |
| 188 | E8062 | 14 | 0.11 | NA | No | NA | NA | NA | NA | NA | NA | NA |
| 189 | E8227 | 12 | 0.11 | NA | No | NA | NA | NA | NA | NA | NA | NA |
| 190 | E8212 | 10 | 0.11 | NA | No | NA | NA | NA | NA | NA | NA | NA |
| 191 | E8087 | 11 | 0.12 | NA | No | NA | NA | NA | NA | NA | NA | NA |
| 192 | E8084 | 10 | 0.12 | NA | No | NA | NA | NA | NA | NA | NA | NA |
| 193 | E8044 | 10 | 0.12 | NA | No | NA | NA | NA | NA | NA | NA | NA |
| 194 | E8066 | 14 | 0.12 | NA | No | NA | NA | NA | NA | NA | NA | NA |
| 195 | E8204 | 9 | 0.12 | NA | No | NA | NA | NA | NA | NA | NA | NA |
| 196 | E8106 | 4 | 0.12 | NA | No | NA | NA | NA | NA | NA | NA | NA |
| 197 | E8211 | 10 | 0.12 | NA | No | NA | NA | NA | NA | NA | NA | NA |
| 198 | E8024 | 11 | 0.12 | NA | No | NA | NA | NA | NA | NA | NA | NA |
| 199 | E8079 | 11 | 0.13 | NA | No | NA | NA | NA | NA | NA | NA | NA |
| 200 | E8018 | 12 | 0.13 | NA | No | NA | NA | NA | NA | NA | NA | NA |
| 201 | E8208 | 9 | 0.13 | NA | No | NA | NA | NA | NA | NA | NA | NA |
| 202 | E8141 | 12 | 0.13 | NA | No | NA | NA | NA | NA | NA | NA | NA |
| 203 | E8000 | 11 | 0.13 | NA | No | NA | NA | NA | NA | NA | NA | NA |
| 204 | E8094 | 11 | 0.13 | NA | No | NA | NA | NA | NA | NA | NA | NA |
| 205 | E8092 | 15 | 0.13 | NA | No | NA | NA | NA | NA | NA | NA | NA |
| 206 | E8224 | 10 | 0.13 | NA | No | NA | NA | NA | NA | NA | NA | NA |
| 207 | E8068 | 11 | 0.14 | NA | No | NA | NA | NA | NA | NA | NA | NA |
| 208 | E8085 | 11 | 0.14 | NA | No | NA | NA | NA | NA | NA | NA | NA |
| 209 | E8140 | 9 | 0.14 | NA | No | NA | NA | NA | NA | NA | NA | NA |
| 210 | E8213 | 9 | 0.14 | NA | No | NA | NA | NA | NA | NA | NA | NA |
| 211 | E8031 | 11 | 0.14 | NA | No | NA | NA | NA | NA | NA | NA | NA |
| 212 | E8136 | 8 | 0.14 | NA | No | NA | NA | NA | NA | NA | NA | NA |
| 213 | E8027 | 14 | 0.14 | NA | No | NA | NA | NA | NA | NA | NA | NA |
| 214 | E8012 | 12 | 0.15 | NA | No | NA | NA | NA | NA | NA | NA | NA |
| 215 | E8206 | 12 | 0.15 | NA | No | NA | NA | NA | NA | NA | NA | NA |
| 216 | E8228 | 12 | 0.15 | NA | No | NA | NA | NA | NA | NA | NA | NA |
| 217 | E8104 | 11 | 0.15 | NA | No | NA | NA | NA | NA | NA | NA | NA |
| 218 | E8061 | 12 | 0.15 | NA | No | NA | NA | NA | NA | NA | NA | NA |
| 219 | E8055 | 11 | 0.15 | NA | No | NA | NA | NA | NA | NA | NA | NA |
| 220 | E8215 | 10 | 0.15 | NA | No | NA | NA | NA | NA | NA | NA | NA |
| 221 | E8005 | 12 | 0.15 | NA | No | NA | NA | NA | NA | NA | NA | NA |
| 222 | E8080 | 11 | 0.16 | NA | No | NA | NA | NA | NA | NA | NA | NA |
| 223 | E8065 | 13 | 0.16 | NA | No | NA | NA | NA | NA | NA | NA | NA |
| 224 | E8078 | 12 | 0.16 | NA | No | NA | NA | NA | NA | NA | NA | NA |
| 225 | E8038 | 13 | 0.16 | NA | No | NA | NA | NA | NA | NA | NA | NA |
| 226 | E8132 | 11 | 0.16 | NA | No | NA | NA | NA | NA | NA | NA | NA |
| 227 | E8145 | 10 | 0.16 | NA | No | NA | NA | NA | NA | NA | NA | NA |
| 228 | E8058 | 11 | 0.17 | NA | No | NA | NA | NA | NA | NA | NA | NA |
| 229 | E8047 | 11 | 0.17 | NA | No | NA | NA | NA | NA | NA | NA | NA |
| 230 | E8022 | 14 | 0.17 | NA | No | NA | NA | NA | NA | NA | NA | NA |
| 231 | E8124 | 14 | 0.17 | NA | No | NA | NA | NA | NA | NA | NA | NA |
| 232 | E8013 | 15 | 0.18 | NA | No | NA | NA | NA | NA | NA | NA | NA |
| 233 | E8121 | 9 | 0.18 | NA | No | NA | NA | NA | NA | NA | NA | NA |
| 234 | E8075 | 7 | 0.18 | NA | No | NA | NA | NA | NA | NA | NA | NA |
| 235 | E8028 | 11 | 0.18 | NA | No | NA | NA | NA | NA | NA | NA | NA |
| 236 | E8111 | 11 | 0.18 | NA | No | NA | NA | NA | NA | NA | NA | NA |
| 237 | E8053 | 11 | 0.19 | NA | No | NA | NA | NA | NA | NA | NA | NA |
| 238 | E8039 | 13 | 0.19 | NA | No | NA | NA | NA | NA | NA | NA | NA |
| 239 | E8089 | 10 | 0.19 | NA | No | NA | NA | NA | NA | NA | NA | NA |
| 240 | E8123 | 7 | 0.19 | NA | No | NA | NA | NA | NA | NA | NA | NA |
| 241 | E8128 | 7 | 0.19 | NA | No | NA | NA | NA | NA | NA | NA | NA |
| 242 | E8105 | 7 | 0.19 | NA | No | NA | NA | NA | NA | NA | NA | NA |
| 243 | E8008 | 12 | 0.19 | NA | No | NA | NA | NA | NA | NA | NA | NA |
| 244 | E8026 | 14 | 0.20 | NA | No | NA | NA | NA | NA | NA | NA | NA |
| 245 | E8120 | 6 | 0.20 | NA | No | NA | NA | NA | NA | NA | NA | NA |
| 246 | E8049 | 10 | 0.20 | NA | No | NA | NA | NA | NA | NA | NA | NA |
| 247 | E8139 | 7 | 0.20 | NA | No | NA | NA | NA | NA | NA | NA | NA |
| 248 | E8137 | 13 | 0.21 | NA | No | NA | NA | NA | NA | NA | NA | NA |
| 249 | E8064 | 12 | 0.21 | NA | No | NA | NA | NA | NA | NA | NA | NA |
| 250 | E8032 | 11 | 0.21 | NA | No | NA | NA | NA | NA | NA | NA | NA |
| 251 | E8050 | 12 | 0.21 | NA | No | NA | NA | NA | NA | NA | NA | NA |
| 252 | E8021 | 12 | 0.21 | NA | No | NA | NA | NA | NA | NA | NA | NA |
| 253 | E8096 | 14 | 0.22 | NA | No | NA | NA | NA | NA | NA | NA | NA |
| 254 | E8220 | 12 | 0.22 | NA | No | NA | NA | NA | NA | NA | NA | NA |
| 255 | E8046 | 10 | 0.22 | NA | No | NA | NA | NA | NA | NA | NA | NA |
| 256 | E8225 | 13 | 0.23 | NA | No | NA | NA | NA | NA | NA | NA | NA |
| 257 | E8010 | 14 | 0.23 | NA | No | NA | NA | NA | NA | NA | NA | NA |
| 258 | E8056 | 11 | 0.23 | NA | No | NA | NA | NA | NA | NA | NA | NA |
| 259 | E8063 | 9 | 0.24 | NA | No | NA | NA | NA | NA | NA | NA | NA |
| 260 | E8069 | 14 | 0.24 | NA | No | NA | NA | NA | NA | NA | NA | NA |
| 261 | E8125 | 10 | 0.24 | NA | No | NA | NA | NA | NA | NA | NA | NA |
| 262 | E8099 | 12 | 0.25 | NA | No | NA | NA | NA | NA | NA | NA | NA |
| 263 | E8054 | 12 | 0.25 | NA | No | NA | NA | NA | NA | NA | NA | NA |
| 264 | E8077 | 11 | 0.26 | NA | No | NA | NA | NA | NA | NA | NA | NA |
| 265 | E8129 | 12 | 0.26 | NA | No | NA | NA | NA | NA | NA | NA | NA |
| 266 | E8109 | 13 | 0.26 | NA | No | NA | NA | NA | NA | NA | NA | NA |
| 267 | E8059 | 12 | 0.27 | NA | No | NA | NA | NA | NA | NA | NA | NA |
| 268 | E8001 | 12 | 0.27 | NA | No | NA | NA | NA | NA | NA | NA | NA |
| 269 | E8025 | 13 | 0.28 | NA | No | NA | NA | NA | NA | NA | NA | NA |
| 270 | E8101 | 9 | 0.28 | NA | No | NA | NA | NA | NA | NA | NA | NA |
| 271 | E8003 | 11 | 0.28 | NA | No | NA | NA | NA | NA | NA | NA | NA |
| 272 | E8144 | 12 | 0.28 | NA | No | NA | NA | NA | NA | NA | NA | NA |
| 273 | E8138 | 10 | 0.29 | NA | No | NA | NA | NA | NA | NA | NA | NA |
| 274 | E8036 | 12 | 0.29 | NA | No | NA | NA | NA | NA | NA | NA | NA |
| 275 | E8205 | 10 | 0.29 | NA | No | NA | NA | NA | NA | NA | NA | NA |
| 276 | E8045 | 10 | **0.30** | NA | No | NA | NA | NA | NA | NA | NA | NA |
| 277 | E8222 | 9 | **0.30** | NA | No | NA | NA | NA | NA | NA | NA | NA |
| 278 | E8015 | 13 | **0.30** | NA | No | NA | NA | NA | NA | NA | NA | NA |
| 279 | E8135 | 12 | **0.32** | NA | No | NA | NA | NA | NA | NA | NA | NA |
| 280 | E8011 | 12 | **0.32** | NA | No | NA | NA | NA | NA | NA | NA | NA |
| 281 | E8037 | 14 | **0.32** | NA | No | NA | NA | NA | NA | NA | NA | NA |
| 282 | E8110 | 12 | **0.33** | NA | No | NA | NA | NA | NA | NA | NA | NA |
| 283 | E8203 | 12 | **0.34** | NA | No | NA | NA | NA | NA | NA | NA | NA |
| 284 | E8131 | 11 | **0.34** | NA | No | NA | NA | NA | NA | NA | NA | NA |
| 285 | E8115 | 13 | **0.35** | NA | No | NA | NA | NA | NA | NA | NA | NA |
| 286 | E8033 | 12 | **0.38** | NA | No | NA | NA | NA | NA | NA | NA | NA |
| 287 | E8051 | 15 | **0.38** | NA | No | NA | NA | NA | NA | NA | NA | NA |
| 288 | E8017 | 13 | **0.38** | NA | No | NA | NA | NA | NA | NA | NA | NA |
| 289 | E8122 | 13 | **0.38** | NA | No | NA | NA | NA | NA | NA | NA | NA |
| 290 | E8097 | 11 | **0.39** | NA | No | NA | NA | NA | NA | NA | NA | NA |
| 291 | E8076 | 11 | **0.40** | NA | No | NA | NA | NA | NA | NA | NA | NA |
| 292 | E8207 | 12 | **0.41** | NA | No | NA | NA | NA | NA | NA | NA | NA |
| 293 | E8081 | 11 | **0.41** | NA | No | NA | NA | NA | NA | NA | NA | NA |
| 294 | E8041 | 15 | **0.43** | NA | No | NA | NA | NA | NA | NA | NA | NA |
| 295 | E8088 | 12 | **0.45** | NA | No | NA | NA | NA | NA | NA | NA | NA |
| 296 | E8083 | 10 | **0.48** | NA | No | NA | NA | NA | NA | NA | NA | NA |
| 297 | E8074 | 13 | **0.48** | NA | No | NA | NA | NA | NA | NA | NA | NA |
| 298 | E8073 | 15 | **0.49** | NA | No | NA | NA | NA | NA | NA | NA | NA |
| 299 | E8107 | 11 | **0.50** | NA | No | NA | NA | NA | NA | NA | NA | NA |
| 300 | E8030 | 12 | **0.53** | NA | No | NA | NA | NA | NA | NA | NA | NA |
| 301 | E8219 | 13 | **0.56** | NA | No | NA | NA | NA | NA | NA | NA | NA |
| 302 | E8043 | 11 | **0.65** | NA | No | NA | NA | NA | NA | NA | NA | NA |
| 303 | E8002 | 12 | **0.70** | NA | No | NA | NA | NA | NA | NA | NA | NA |
| 304 | E8072 | 13 | **0.71** | NA | No | NA | NA | NA | NA | NA | NA | NA |
| 305 | E8020 | 13 | **0.79** | NA | No | NA | NA | NA | NA | NA | NA | NA |
| 306 | E8016 | 15 | **0.84** | NA | No | NA | NA | NA | NA | NA | NA | NA |
| 307 | E8035 | 12 | **0.91** | NA | No | NA | NA | NA | NA | NA | NA | NA |
| 308 | E8217 | 14 | **1.04** | NA | No | NA | NA | NA | NA | NA | NA | NA |
| 309 | E8112 | 10 | **1.07** | NA | No | NA | NA | NA | NA | NA | NA | NA |
| 310 | E8095 | 14 | **1.08** | NA | No | NA | NA | NA | NA | NA | NA | NA |
| 311 | E8114 | 16 | **1.13** | NA | No | NA | NA | NA | NA | NA | NA | NA |
| 312 | E8048 | 11 | **1.24** | NA | No | NA | NA | NA | NA | NA | NA | NA |
| 313 | 3714 | 12 | 0.29 | NA | No | NA | NA | NA | NA | NA | NA | NA |
| 314 | 3715 | 13 | **0.33** | NA | No | NA | NA | NA | NA | NA | NA | NA |
| 315 | 3709 | 15 | **0.37** | NA | No | NA | NA | NA | NA | NA | NA | NA |
| 316 | 3712 | 13 | **0.38** | NA | No | NA | NA | NA | NA | NA | NA | NA |
| 317 | 3706 | nd | **0.66** | NA | No | NA | NA | NA | NA | NA | NA | NA |
| 318 | 3711 | 24 | **0.67** | NA | No | NA | NA | NA | NA | NA | NA | NA |

**HHC: Household contacts; †SC: School children; ‡NA: Not available; nd: not determined*
